# Correction: Mora-Palazuelos et al. The Role of ncRNAs in the Immune Dysregulation of Preeclampsia. *Int. J. Mol. Sci.* 2023, *24*, 15215

**DOI:** 10.3390/ijms262311334

**Published:** 2025-11-24

**Authors:** Carlos Mora-Palazuelos, Carlos Esteban Villegas-Mercado, Mariana Avendaño-Félix, Erik Lizárraga-Verdugo, José Geovanni Romero-Quintana, Jorge López-Gutiérrez, Saúl Beltrán-Ontiveros, Mercedes Bermúdez

**Affiliations:** 1 Health Sciences Research and Teaching Center, Autonomous University of Sinaloa, Culiacan 80010, Sinaloa, Mexico; carlospalazuelos@uas.edu.mx (C.M.-P.); eriklizarraga@uas.edu.mx (E.L.-V.); saul.beltran@uas.edu.mx (S.B.-O.); 2 Faculty of Dentistry, Autonomous University of Chihuahua, Chihuahua 31110, Chihuahua, Mexico; cmercado@uach.mx; 3 Faculty of Dentistry, Autonomous University of Sinaloa, Culiacan 80010, Sinaloa, Mexico; marianaavendano@uas.edu.mx; 4 Faculty of Chemical and Biological Sciences, Autonomous University of Sinaloa, Culiacan 80010, Sinaloa, Mexico; geovanniromero@uas.edu.mx; 5 Faculty of Biology, Autonomous University of Sinaloa, Culiacan 80010, Sinaloa, Mexico; doctorjorgelopez@uas.edu.mx

The journal’s Editorial Office and Editorial Board are jointly issuing a resolution and removal of the Journal Notice linked to this article [1], as well as an update to the original publication. Following concerns raised about the integrity of the peer-review, the Editorial Office has conducted a post-publication peer-review of this article. This process included the recruitment of a new independent reviewer and was supervised by an Editorial Board member to ensure full compliance with MDPI’s Editorial Process (https://www.mdpi.com/editorial_process).

As a result of this process, the Editorial Board member and the authors have agreed to remove Reviewer 1 report and upload the new one to the peer review record (https://www.mdpi.com/1422-0067/24/20/15215/review_report).

With this update, the Academic Editor is satisfied that the Editorial Process relating to this article has been completed as per MDPI’s Editorial Process policy. The Editorial Office would like to thank the authors for their collaboration during this process.

## References

[1] Mora-Palazuelos C., Villegas-Mercado C.E., Avendaño-Félix M., Lizárraga-Verdugo E., Romero-Quintana J.G., López-Gutiérrez J., Beltrán-Ontiveros S., Bermúdez M. (2023). The Role of ncRNAs in the Immune Dysregulation of Preeclampsia. Int. J. Mol. Sci..

